# Editorial: Innovations in microbiome applications for health-promoting and sustainable food systems

**DOI:** 10.3389/fmicb.2024.1538798

**Published:** 2025-01-03

**Authors:** Yiannis Kourkoutas, Nikolaos Kopsahelis, Antonios Koutelidakis, Andreas G. Tzakos

**Affiliations:** ^1^Department of Molecular Biology & Genetics, Democritus University of Thrace, Alexandroupolis, Greece; ^2^Department of Food Science & Technology, Ionian University, Kefalonia, Greece; ^3^Department of Food Science & Nutrition, Aegean University, Myrina, Greece; ^4^Department of Chemistry, University of Ioannina, Ioannina, Greece

**Keywords:** functional foods, plant extracts, agri-food wastes, biorefineries, novel functional foods regulations

Innovations in microbiome research are pivotal for advancing health-promoting and sustainable food systems, aligning with key priorities outlined in bioeconomy strategies, such as the European Green Deal. Central to food innovation is the development of novel foods enriched with beneficial microorganisms and bioactive compounds, designed to enhance their functional properties. This objective is a core focus of the Infrastructure of Microbiome Applications in Food Systems-FOODBIOMES (www.foodbiomes.eu), a Greek research infrastructure committed to microbiome applications to transform food systems and drive progress in sustainability and human health.

Hence, this Research Topic explores the transformative role of microbiome innovations in fostering health-promoting and sustainable food systems. It brings together cutting-edge research on the design and application of microbiome-based solutions, focusing on their potential to enhance food functionality, address environmental challenges, and combat health issues, such as gut dysbiosis, aging, antibiotic resistance, atopic dermatitis, and endometritis.

Chen et al. selected two strains of lactic acid bacteria for fermenting bamboo shoots. *Lactiplantibacillus plantarum* R1 exhibited prominent potential probiotic properties (including gastrointestinal condition tolerance, adhesion ability, antimicrobial ability, and antibiotic resistance), while the strain *Levilactobacillus brevis* R2 was able to produce a high content of γ-aminobutyric acid (GABA). The synergistic inoculation of both strains during bamboo shoot fermentation led to a remarkable increase in GABA content, surpassing that of naturally fermented bamboo shoots by more than 4.5 times and outperforming mono-inoculated fermentation. Simultaneously, the nitrite content was maintained within the recommended levels (5.96 ± 1.81 mg/kg). In addition, inoculated fermented bamboo shoots exhibited an increased crude fiber content and reduced fat content. Thus, the safe and rapid fermentation of bamboo shoots may lay the groundwork for the development of functional vegetable products enriched with GABA.

Gut microbiota dysbiosis is a serious risk factor for several gastric and systemic diseases. Recently, available preclinical evidence suggests that the probiotic bacterium *Lactiplantibacillus plantarum* (LP) may influence the aging process *via* modulation of the gut microbiota. The review authored by Gupta et al. summarized compelling evidence of LP's potential effect on aging hallmarks, such as oxidative stress, inflammation, DNA methylation, and mitochondrial dysfunction. In short, LP cell constituents exert considerable antioxidant potential that may reduce ROS levels directly, restore gut microbiota, facilitate a healthy intestinal milieu, and accelerate multi-channel communication via signaling factors, such as SCFA and GABA. These signaling factors further activated the transcription factor Nrf2 and reduced oxidative damage. The authors concluded that LP supplementation may be an effective approach to managing aging and associated health risks.

In an era increasingly defined by the challenge of antibiotic resistance, the study by Huang et al. offered groundbreaking insights into the antibacterial properties of two distinct *Lactiplantibacillus plantarum* strains (TE0907 and TE1809), hailing from the unique ecosystem of *Bufo gargarizans*, as it uniquely focused on elucidating the intricate components and mechanisms that empower these strains with their notable antibacterial capabilities. The research used a multi-omics approach, including agar diffusion tests to assess antibacterial efficacy and adhesion assays with HT-29 cells to understand the preliminary mechanisms. Additionally, gas chromatography-mass spectrometry (GC-MS) was used to analyze the production of organic acids, while whole-genome sequencing was used to identify genes linked to the biosynthesis of antibiotics and bacteriocin-encoding domains. The comparative analysis highlighted the exceptional antibacterial efficacy of both strains. A pivotal discovery was the synthesis of acetic acid in both strains, linking its abundance to their antimicrobial efficiency. Genomic exploration uncovered a diverse range of elements involved in the biosynthesis of antibiotics, similar to tetracycline and vancomycin, and potential regions encoding bacteriocins, including enterolysin, and plantaricin. The findings underscored the strains' extensive biochemical and enzymatic armamentarium, offering valuable insights into their role in antagonizing enteric pathogens for potential clinical deployment in safeguarding animal gut health, thereby enriching our understanding of the role of probiotic bacteria in the realm of antimicrobial intervention.

Traditional fermented foods have long been recognized for their numerous health benefits along with their potential to aid in the treatment of GI disorders. In this study, high-throughput sequencing using the Illumina MiSeq platform was used to investigate the microbiome communities of rice-based fermented beverages consumed by ethnic tribes in southern Assam, namely the Zeme Naga, Dimasa Kachari, Hmar, Karbi, and Tea tribes (Yumnam et al.). The fermented rice-based beverages were highly predominated by *Firmicutes, Bacteroides, Proteobacteria*, and *Actinobacteria*, exhibiting the highest relative abundance across all tribes. At the genus level, the significant abundance of *Pediococcus, Lactobacillus, Bacillus, Leuconostoc, Acetobacter, Staphylococcus, Delftia, Erwinia, Klebsiella*, and *Chryseobacterium* was found among these ethnic tribes. Understanding the fermented food microbiome is expected to provide insights into the relationships between microbial communities and their effect on the health of humans among the tribes.

As the effects of fructooligosaccharides (FOS) on atopic dermatitis (AD) have not been fully determined, Kim et al. studied the impact of 1-kestose on AD skin beyond the clinical aspects, including the modulation of the epidermal skin barrier and lipid profiles, along with the skin microbiome in a randomized, double-blind, placebo-controlled trial using children with AD aged 24 months to 17 years that received either advanced FOS containing 4.25 g of 1-kestose or a placebo (maltose) for 12 weeks. The SCORAD and itching scores were reduced in patients treated with both FOS and maltose. Sleep disturbance was improved only in the FOS group. The FOS group revealed a decreased proportion of linoleic acid (18:2) esterified omega-hydroxy-ceramides (EOS-CERs) with amide-linked shorter chain fatty acids (C28 and C30), along with an increased proportion of EOS-CERs with longer chain fatty acids (C32). The authors concluded that FOS may be beneficial in alleviating itching and sleep disturbances and improving skin barrier function in children with AD.

Since endometritis occurs frequently in humans and animals and can negatively affect fertility and cause preterm parturition syndrome, Hagihara et al. evaluated the anti-inflammatory effects of orally administered *Clostridium butyricum* on uterine tissues. Additionally, uterine microbiome and lipid metabolome analyses were performed to determine the underlying mechanisms. Although it is known that orally administered *Clostridium butyricum*, a butyrate-producing gram-positive anaerobe, can exhibit anti-inflammatory effects, the precise mechanism by which *Clostridium butyricum* attenuates endometritis remains unclear. Oral administration of *Clostridium butyricum* altered the uterine microbiome, inducing the proliferation of *Lactobacillus* and *Limosilactobacillus* species. Additionally, oral administration of *Clostridium butyricum* resulted in the upregulation of some lipid metabolites, such as the ω-3 polyunsaturated fatty acid resolvin D5 in uterine tissues, and resolvin D5 showed anti-inflammatory effects. However, the anti-inflammatory effects induced by orally administered *Clostridium butyricum* were significantly diminished when G protein-coupled receptor 120 was deleted or 15-lipoxygenase activity was inhibited. In conclusion, *Clostridium butyricum* in the gut had anti-inflammatory effects on uterine tissues through alterations in the uterine microbiome and lipid metabolism.

In summary, this Research Topic highlights the pivotal role of microbiome innovations in tackling global health and sustainability challenges. The research presented demonstrates the broad applications of microbiomes, from enhancing the functional properties of foods to advancing targeted health interventions for both humans and animals. The key themes surveyed include breakthroughs in fermentation technologies, the development of functional food ingredients and final products, and the exploration of microbiome-based antibiotic resistance strategies. Together, this Research Topic of studies advances our understanding and application of the microbiome, bridging the gap between cutting-edge research and practical solutions, while promoting progress toward sustainable food systems and health-focused technologies.

